# A new accuracy measure based on bounded relative error for time series forecasting

**DOI:** 10.1371/journal.pone.0174202

**Published:** 2017-03-24

**Authors:** Chao Chen, Jamie Twycross, Jonathan M. Garibaldi

**Affiliations:** School of Computer Science, University of Nottingham, Nottingham, United Kingdom; Tianjin University, CHINA

## Abstract

Many accuracy measures have been proposed in the past for time series forecasting comparisons. However, many of these measures suffer from one or more issues such as poor resistance to outliers and scale dependence. In this paper, while summarising commonly used accuracy measures, a special review is made on the symmetric mean absolute percentage error. Moreover, a new accuracy measure called the Unscaled Mean Bounded Relative Absolute Error (UMBRAE), which combines the best features of various alternative measures, is proposed to address the common issues of existing measures. A comparative evaluation on the proposed and related measures has been made with both synthetic and real-world data. The results indicate that the proposed measure, with user selectable benchmark, performs as well as or better than other measures on selected criteria. Though it has been commonly accepted that there is no single best accuracy measure, we suggest that UMBRAE could be a good choice to evaluate forecasting methods, especially for cases where measures based on geometric mean of relative errors, such as the geometric mean relative absolute error, are preferred.

## Introduction

Forecasting has always been an attractive research area since it plays an important role in daily life. As one of the most popular research domains, time series forecasting has received particular concern from researchers [1–5]. Many comparative studies have been conducted with the aim of identifying the most accurate methods for time series forecasting [6]. However, research findings indicate that the performance of forecasting methods varies according to the accuracy measure being used [7]. Various accuracy measures have been proposed as the best to use in the past decades. However, many of these measures are not generally applicable due to issues such as being infinite or undefined under certain circumstances, which may produce misleading results. The criteria required for accuracy measures have been explicitly addressed by Armstrong and Collopy [6] and further discussed by Fildes [8] and Clements and Hendry [9]. As discussed, a good accuracy measure should provide an informative and clear summary of the error distribution. The criteria should also include reliability, construct validity, computational complexity, outlier protection, scale-independency, sensitivity to changes and interpretability. It has been suggested by many researchers that no single measure can be superior to all others in these criteria [6, 10, 11].

The evolution of accuracy measures can be seen through the measures used in the major comparative studies of forecasting methods. Root Mean Square Error (RMSE) and Mean Absolute Percentage Error (MAPE) can be considered as the very early and most popular accuracy measures. They were the primary measures used in the original M-Competition [12]. Despite well-known issues such as their high sensitivity to outliers, they are still being widely used [13–15]. When using these accuracy measures, errors which are small and appear to be good, such as 0.1 by RMSE and 1% by MAPE, can often be obtained. Wei et al. [16] employed RMSE as the performance indicator in their research on stock price forecasting. The average error obtained was 84 and it was claimed to be superior to some other previous models. However, without comparison, the error 84 as a number is not easy to interpret. In fact, the average fluctuation of stock indices used was 83 which is smaller than the error of their proposed model. A similar case can be found regarding MAPE. Esfahanipour and Aghamiri [17] proposed a model with an error of 1.3%, which appears to be good. Yet, this error was larger than the average daily fluctuation of the stock price, which was approximately 1.2%. The poor interpretation here is mainly due to the lack of comparable benchmark used by the accuracy measure.

Armstrong and Collopy [6] recommended the use of relative absolute errors as a potential solution to the above issue. Accuracy measures based on relative errors, such as Mean Relative Absolute Error (MRAE), can provide a better interpretation of how good the evaluated forecasting method perform compared to the benchmark method. However, when the benchmark error is small or equal to zero, the relative error could become extremely large or infinite. This may lead to an undefined mean or at least a distortion of the result. Thus, Armstrong and Collopy suggested a method named ‘winsorizing’ to overcome this problem by trimming extreme values. However, this process will also add some complexity to the calculation and an appropriate trimming level has to be specified [18].

Similarly, MAPE also has the issue of being infinite or undefined due to zeros in the denominator [19]. The symmetric mean absolute percentage error (sMAPE) was first proposed by Armstrong [20] as a modified MAPE which could be a simple way to fix the issue. It was then used in the M3-Competition as an alternative primary measure to MAPE [7]. However, Goodwin and Lawton [21] pointed out that sMAPE is not as symmetric as its name suggested. In fact, it gave more penalties to under-estimates more than to over-estimates. Thus, the use of sMAPE in the M3-Competition was widely criticized by researchers later [22]. In an unpublished working paper, Chen and Yang [23] defined a modified sMAPE, called msMAPE, by adding an additional component to the denominator of sMAPE. The added component can efficiently avoid the inflation of sMAPE caused by zero-valued observations. However, this does not address the issue of asymmetry for sMAPE.

Hyndman and Koehler [18] proposed Mean Absolute Scaled Error (MASE) as a generally applicable measurement of forecasting accuracy without the problems seen in the other accuracy measures. However, this measure can still be dominated by a single large error, though infinite and undefined values have been well avoided for most cases [24]. Davydenko and Fildes [24] proposed an altered version of MASE, the average relative MAE (AvgRelMAE), which uses the geometric mean to average the relative efficiencies of adjustments across time series. Although the geometric mean is appropriate for averaging benchmark ratios [25], the appropriateness of AvgRelMAE still depends on its component measure RelMAE for each time series.

In this paper, a new accuracy measure is proposed to address the issues mentioned above. Specifically, by introducing a newly defined bounded relative absolute error, the new measure can address the asymmetric issue of sMAPE while maintaining its other properties, such as scale-independence and outlier resistance. Further, we believe that the new measure improves the interpretability based on relative errors with a selectable benchmark than sMAPE which uses the percentage errors based on the observation values. Given that [6] claimed that measures based on relative errors are the most reliable, we believe our measure is reliable in this sense.

## Review of accuracy measures

Many accuracy measures have been proposed to evaluate the performance of forecasting methods during the past couple of decades. A table of most commonly used measures were listed in the review of 25 years of time series forecasting [1]. There was also a thorough review on accuracy measures by Hyndman and Koehler [18]. In this section, we mainly focus on new insights or new measures that have been introduced since 2006.

For a time series with *n* observations, let *Y*_*t*_ denote the observation at time *t* and *F*_*t*_ denote the forecasts of *Y*_*t*_. Then the forecasting error *e*_*t*_ can be defined as (*Y_t_–F_t_*). Let $e_{t}^{*}$ denote the forecasting error at time *t* obtained by some benchmark method. That means $e_{t}^{*}=\left(Y_{t}-F_{t}^{*}\right)$, where $F_{t}^{*}$ is the forecast at time *t* by the benchmark method.

### Scale-dependent measures

The measures based on absolute or squared errors are also known as scale-dependent measures since their scale depends on the scale of the data. They are useful in comparing forecasting methods on the same set of data. However, they should not be used across data sets that are on different scales. The most commonly used scale-dependent measures are Mean Absolute Error (MAE), Mean Squared Error (MSE) and RMSE:

$$\begin{array}{ccc} MAE & = & \frac{1}{n}\sum_{t=1}^{n}\left|e_{t}\right| \end{array}$$ (1)

$$\begin{array}{ccc} MSE & = & \frac{1}{n}\sum_{t=1}^{n}e_{t}^{2} \end{array}$$ (2)

$$\begin{array}{ccc} RMSE & = & \sqrt{MSE} \end{array}$$ (3)

MAE had been cited in the very early forecasting literature as a primary measure of performance for forecasting models [26]. As shown in Eq 1, MAE directly calculates the arithmetic mean of absolute errors. Hence, it is very easy to compute and to understand. However, it may produce biased results when extremely large outliers exist in data sets. Specifically, even a single large error can sometimes dominate the result of MAE.

MSE, which calculates the arithmetic mean of squared errors, was used in the first M-Competition [12]. However, its use was widely criticized later as inappropriate [6, 27]. MSE is more vulnerable to outliers since it gives extra weight to large errors. Also, the squared errors are on different scale from the original data. Thus, RMSE, which is the squre root of MSE, is often preferred to MSE as it is on the same scale as the data. However, RMSE is also sensitive to forecasting outliers [28].

### Percentage-based measures

To be scale-independent, a common approach is to use percentage errors based on observation values. Two example measures based on percentage errors are MAPE and sMAPE defined as:

$$\begin{array}{ccc} MAPE & = & \frac{1}{n}\sum_{t=1}^{n}\frac{|e_{t}|}{|Y_{t}|} \end{array}$$ (4)

$$\begin{array}{ccc} sMAPE & = & \frac{1}{n}\sum_{t=1}^{n}\frac{2\cdot |e_{t}|}{|Y_{t}\left|+\right|F_{t}|} \end{array}$$ (5)

It should be noted that absolute values are used in the denominator of sMAPE defined in this paper. This definition is different but equivalent to the definition in Makridakis [10] and Makridakis and Hibon [7] when forecasts and actual values are all non-negative. The absolute values in the denominator can avoid negative sMAPE as pointed out by Hyndman and Koehler [18].

MAPE was used as one of the major accuracy measures in the original M-Competition [12]. However, the percentage errors could be excessively large or undefined when the target time series has values close to or equal to zero [19]. Moreover, Armstrong [20] pointed out that MAPE has a bias favouring estimates that are below the actual values. This was illustrated by extremes: *“a forecast of 0 can never be off by more than 100%, but there is no limit to errors on the high side”*. Makridakis [10] discussed the asymmetric issue of MAPE with another example which involves two forecasts on different actual values. However, we believe that the example by Makridakis is beyond the idea of Armstrong in 1985. To our understanding, we believe that the assumption concerning the asymmetric issue of MAPE described by Armstrong [20] is: i), the estimates are non-negative while the actual value is positive; ii) the forecasting range is asymmetric that 0 is the lower bound for lower estimates while there is no upper bound for upper estimates; iii), errors for lower estimates and upper estimates should be symmetric (an extreme case: 0 as the worst lower estimate should have the same absolute error as the worst upper estimate which is infinite).

sMAPE can produce symmetric errors in the asymmetric forecasting range as stated in the above assumption. However, it is more natural to consider the symmetric property in a symmetric forecasting range for lower and upper estimates. Thus, sMAPE was widely criticized as an asymmetric measure [21, 22]. Regardless of the asymmetric issue, an advantage of sMAPE is that it does not have the issue of MAPE from being excessively large or infinite. Also, due to the error bounds defined, sMAPE is more resistant to outliers since it gives less significance to outliers compared to other measures which do not have bounds for errors.

### Relative-based measures

Another approach for accuracy measures to be scale-independent is to use relative errors based on the errors produced by a benchmark method (e.g. the naïve method). The most commonly used such measures are MRAE and the geometric mean relative absolute error (GMRAE):

$$\begin{array}{ccc} MRAE & = & \frac{1}{n}\sum_{t=1}^{n}\left|\frac{e_{t}}{e_{t}^{*}}\right| \end{array}$$ (6)

$$\begin{array}{ccc} GMRAE & = & {\left(\prod_{t=1}^{n}\left|\frac{e_{t}}{e_{t}^{*}}\right|\right)}^{\frac{1}{n}}=\;exp\left(\frac{1}{n}\sum_{t=1}^{n}ln\left(\frac{e_{t}}{e_{t}^{*}}\right)\right) \end{array}$$ (7)

MRAE can provide a clearer intuition of the performance improvement compared to the benchmark method. However, MRAE has a similar limitation as MAPE, in that it can also be excessively large or undefined, when $e_{t}^{*}$ is close to or equal to zero.

GMRAE is favoured since it is generally acknowledged that the geometric mean is more appropriate for averaging relative quantities than the arithmetic mean [6, 8]. According to an alternative representation of GMRAE shown above in Eq 7, a key step for calculating GMRAE is to make an arithmetic mean of log-scaled error ratios. This makes GMRAE more resistant to outliers compared to MRAE which uses the arithmetic mean of original error ratios. However, GMRAE is still sensitive to outliers. More specifically, GMRAE can be dominated by not only a single large outlier, but also an extremely small error close to zero. This is because there is neither upper bound nor lower bound for the log-scaled error ratios used by GMRAE. Also, it should also be noticed that zero errors, both in *e*_*t*_ and $e_{t}^{*}$, have to be excluded from the analysis. Thus, GMRAE may not be sufficiently informative.

Rather than use the average of relative errors, one can also use the relative of average errors obtained by a base measure. For example, when the base measure is RMSE, then relative RMSE (RelRMSE) is defined as:

$$\begin{array}{c} RelRMSE=\frac{RMSE}{RMSE*} \end{array}$$ (8)

RelRMSE is a commonly used measure proposed by Armstrong and Collopy [6] where RMSE^*^ denotes the *RMSE* produced by a benchmark method. Similar measures, such as RelMAE and RelMAPE, can be easily defined. They are also called relative measures. An advantage of relative measures is their interpretability [18]. However, the performance of relative measures is restricted by the component measure. For example, RelMAPE is also undefined when MAPE is undefined. Further, RelMAPE can also be easily dominated by extreme large outliers since MAPE is not resistant to outliers. Thus, it makes no sense to compute RelMAPE if MAPE, as the component, is skewed.

Another disadvantage of relative measures is that they are only available when there are several forecasts on the same series [18]. As a related idea of relative measures, MASE does not have the above issue. It is defined as:

$$\begin{array}{c} MASE=\frac{1}{n}\sum_{t=1}^{n}\frac{\left|e_{t}\right|}{MAE^{**}} \end{array}$$ (9)

In MASE, the absolute error |*e*_*t*_| for each observation is scaled by the average *in-sample* error MAE^**^ produced a benchmark method (e.g. one-step naïve method, or seasonal naïve method for seasonal data). Thus, MASE will not produce infinite or undefined values except in the irrelevant case where all historical data are equal. However, MASE is still vulnerable to outliers [24]. Moreover, it has to be assumed that the period-to-period difference of the time series is stationary, so that the scaling factor is a consistent estimator of the scale of the series.

For comparisons of forecasting methods on multiple time series, MASE is equivalent to the weighted arithmetic mean of relative MAEs [24]:

$$\begin{array}{ccc} MASE=\frac{1}{N}\sum_{i=1}^{m}n_{i}r_{i}, & & r_{i}=\frac{MAE_{i}}{MAE_{i}^{**}} \end{array}$$ (10)

where *m* denotes the number of time series, *n*_*i*_ denotes the number of observations for the *i*^*th*^ time series and $N=\sum_{i=1}^{m}n_{i}$. As pointed out by Davydenko and Fildes [24], using the arithmetic mean of MAE ratios introduces a bias towards overrating the accuracy of a benchmark method. They proposed the measure AvgRelMAE as an alternative to MASE, based on the geometric mean to average the scaled quantities.

$$\begin{array}{ccc} AvgRelMAE={\left(\prod_{i=1}^{m}r_{i}^{n_{i}}\right)}^{\frac{1}{N}}, & & r_{i}=\frac{MAE_{i}}{MAE_{i}^{*}} \end{array}$$ (11)

It should be noticed that AvgRelMAE uses *out-of-sample* $MAE_{i}^{*}$ as the scaling factor while MASE uses *in-sample* $MAE_{i}^{**}$. Though AvgRelMAE was shown to have many advantages such as interpretability and robustness [24], it still has the same issue with MASE since they are based on RelMAE. As mentioned above, the accuracy of RelMAE is constrained by the accuracy of MAE. Since MAE can be dominated by extreme outliers, the MAE ratio *r*_*i*_ does not necessarily represent an advisable comparison of forecasting methods based on the errors of the majority of forecasts for the *i*^*th*^ time series.

## A new accuracy measure

The criteria for a useful accuracy measure have been explicitly addressed in the literature [6, 8, 9, 11]. As reviewed in the previous Section, many measures have been proposed with various advantages and disadvantages. However, most of these measures suffer from one or more issues. In this section, we propose a new accuracy measure which adopts the advantages of other measures such as sMAPE and MRAE without having their common issues. Specifically, the proposed measure is expected to have the following properties: (i) Informative: it can provide an informative result without the need to trim errors; (ii) Resistant to outliers: it can hardly be dominated by a single forecasting outlier; (iii) Symmetric: over estimates and under estimates are treated fairly; (iv) Scale-independent: it can be applied to data sets on different scales; (v) Interpretability: it is easy to understand and can provide intuitive results.

It has been mentioned above in the review that sMAPE is resistant to outliers due to bounded error defined. We would like to propose a new measure in a similar fashion to sMAPE without its issues. Since relative errors are more general than percentage errors in providing intuitive results, we use the Relative Absolute Error (RAE) as the base to derive our new measure.

$$\begin{array}{ccc} & & RAE=\frac{|e_{t}|}{|e_{t}^{*}|} \end{array}$$ (12)

Since RAE has no upper bound, it can be excessively large or undefined when $|e_{t}^{*}|$ is small or equal to zero. This issue can be easily addressed by adding a |*e*_*t*_| to the denominator of RAE, which introduces a bounded RAE (BRAE):

$$\begin{array}{ccc} & & BRAE=\frac{|e_{t}|}{|e_{t}\left|+\right|e_{t}^{*}|} \end{array}$$ (13)

In BRAE, the added |*e*_*t*_| can ensure that the denominator will be no less than the numerator. It means BRAE will have a maximum error of 1 while the minimum error is 0 when |*e*_*t*_| is equal to zero. Due to the upper bound of BRAE, an accuracy measure based on BRAE will be more resistant to forecasting outliers. It can be noticed that the asymmetric issue of sMAPE has also been addressed in BRAE by adding a |*e*_*t*_| rather than a |*F*_*t*_| to the denominator. Also, a measure based on BRAE is more appropriate than sMAPE for intermittent demand data which have many zero-valued observations. To avoid the issue of being undefined, BRAE is defined to be 0.5 for the special case when |*e*_*t*_| and $|e_{t}^{*}|$ are both equal to zero.

In practice, the one-step naïve method is a commonly used benchmark where $e_{t}^{*}=Y_{t-1}-Y_{t}$. However, it should be noticed that the naïve method is not necessarily an effective benchmark. For example, when most forecasting methods can generally produce much smaller errors than the naïve method, BRAE will have the same issue as percentage error based measure stated above. Thus, it is preferable to use a properly competitive method as a benchmark, such that a value of around 0.5 is obtained by BRAE.

Based on BRAE, a measure called Mean Bounded Relative Absolute Error (MBRAE) can be defined as:

$$\begin{array}{c} MBRAE=mean\left(BRAE\right)=\frac{1}{n}\sum_{t=1}^{n}\frac{|e_{t}|}{|e_{t}\left|+\right|e_{t}^{*}|} \end{array}$$ (14)

Though MBRAE is adequate to compare forecasting methods, it is a scaled error that cannot be directly interpreted as a normal error ratio reflecting the error size. In fact, the process of calculating GMRAE also contains a mean of log-scaled error ratio which is not easily interpretable. But this issue is addressed by converting the log-scaled error to a normal ratio with the exponential function. Similarly, a transformation can be made to MBRAE to obtain a more interpretable measure which is termed the unscaled MBRAE (UMBRAE):

$$\begin{array}{ccc} UMBRAE & = & \frac{MBRAE}{1-MBRAE} \end{array}$$ (15)

With UMBRAE, the performance of a proposed forecasting method can be easily interpreted, in terms of the average relative absolute error based on BRAE, as follows: when *UMBRAE* is equal to 1, the proposed method performs roughly the same as the benchmark method; when *UMBRAE* < 1, the proposed method performs roughly (1−*UMBRAE*)*100% better than the benchmark method; when *UMBRAE* > 1, the proposed method is roughly (*UMBRAE*−1)*100% worse than the benchmark method.

In general, UMBRAE is informative without the need to trim extreme errors. At the same time, based on the bounded errors, UMBRAE is resistant to outliers. It is also symmetric and obviously scale-independent. The benchmark used by UMBRAE is selectable where the naïve method can be easily applied. A competitive benchmark is preferable to obtain more intuitive results. To the best of our knowledge, UMBRAE has not been proposed before. We suggest it as a generally applicable accuracy measure for time series forecasting. UMBRAE would be particularly useful for the cases where the performance of forecasting methods are not expected to be dominated by forecasting outliers.

## Evaluation and results

In this section, the performance of UMBRAE is evaluated. The naïve method is used as the benchmark for UMBRAE. Properties such as reliability and sensitivity have been well investigated in the study by Armstrong and Collopy [6]. In their study, MAPE and MRAE have been assessed to be acceptable in terms of reliability and good in terms of sensitivity. In fact, these properties, especially reliability, cannot be easily examined. For example, in the reliability tests, if forecasting methods are expected to have the same rankings when they are evaluated by a reliable accuracy measure, these forecasting methods themselves have to perform stably on different time series. It is difficult to find such forecasting methods in the real world. Thus, these properties are not examined in our study. Instead, it is assumed that UMBRAE, based on relative errors, will also be reliable and sensitive to error changes. Consequently, our evaluation will be mainly focused on the expected properties mentioned in the previous Section. To make comparisons, other common measures mentioned in the review Section are also examined in our evaluation. Comparisons are firstly made with synthetic time series to specifically examine the required properties. Then the M3-Competition data with 3003 time series [7] are used to demonstrate how these measures perform with real-world data.

### Evaluation with synthetic data

Three groups of synthetic time series data are used in the comparative study. These synthetic data are not designed to be representative of real-world data. Rather, they are selected to clearly show the drawbacks of accuracy measures in terms of the required properties. In the synthetic evaluations, the average one-step naïve error is used to scale errors for MASE.

**Fig 1:**
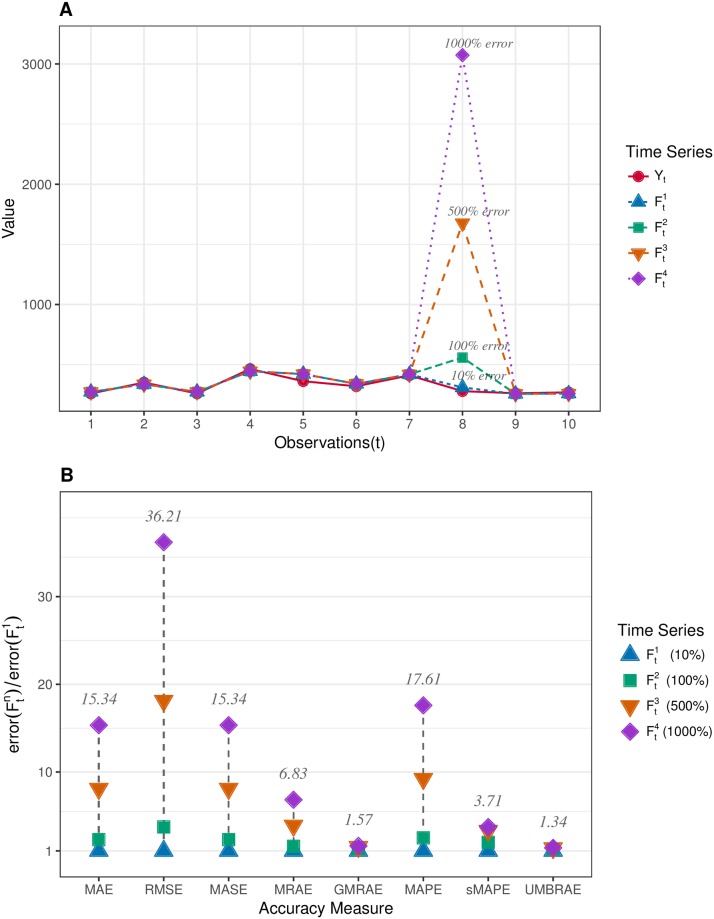
Evaluation on the resistance of accuracy measures to a single forecasting outlier. A: Synthetic time series data where *Y*_*t*_ is the target series and $F_{t}^{n}$ are forecasts. The only difference between $F_{t}^{n}$ is their forecasts on the observation *Y*_8_. B: Results of single forecasting outlier evaluation, which shows UMBRAE is less sensitive than other measures to a single forecasting outlier.

One of the most desired properties of an accuracy measure is the ability to resist outliers. Thus, the first group of synthetic data is made to examine whether the accuracy measure is resistant to a single forecasting outlier. As shown in Fig 1, *Y*_*t*_ is the objective time series with 10 observations, which are randomly generated under the normal distribution (mean = 300, sd = 100). $F_{t}^{n}$ is the forecasting series of *Y*_*t*_. Specifically, $F_{t}^{1}$ does not have obvious forecasting outlier and its forecasting errors measured by MAPE are approximately 10%. The other three forecasts are the same as $F_{t}^{1}$ except that they all have a forecasting outlier for the eighth observation. Though occasionally occurring large errors should also be considered in evaluating the performance of a forecasting method, it is assumed that a single large outlier should not affect the whole performance significantly. However, the results in Fig 1 shows that the errors reported by some accuracy measures have been significantly dominated by the single forecasting outlier. The worst is RMSE where its error for $F_{t}^{4}$ has become approximately 36 times larger than its error for $F_{t}^{1}$. Though MASE has been scaled from MAE, it in fact performs the same as MAE in dealing with the forecasting outlier. The errors given by MAE and MASE for $F_{t}^{4}$ have both been distorted to be about 15 times larger than for $F_{t}^{1}$. In contrast, sMAPE, GMRAE and UMBRAE are less sensitive to this single forecasting outlier. UMBRAE reports the smallest differences for the four time series.

**Fig 2:**
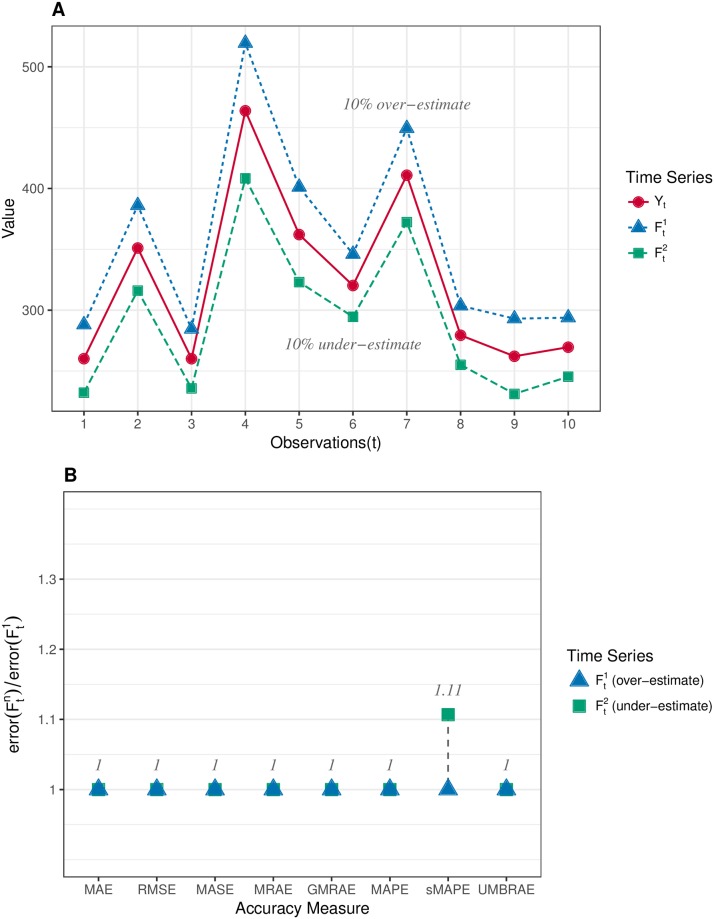
Evaluation on the symmetry of accuracy measures to over-estimates and under-estimates. A: Synthetic time series data where *Y*_*t*_ is the target series and $F_{t}^{n}$ are forecasts. $F_{t}^{1}$ makes a 10% over-estimate to all observations of *Y*_*t*_, while $F_{t}^{2}$ makes a 10% under-estimate. B: Results of symmetric evaluation, which shows UMBRAE and all other accuracy measures except sMAPE are symmetric.

The second group of time series data is created to evaluate whether over-estimates and under-estimates are treated ‘fairly’ by the accuracy measures. As presented in Fig 2, *Y*_*t*_ is the same time series as which was used in the single forecasting outlier resistance evaluation. In this scenario, $F_{t}^{1}$ makes a 10% over-estimate error to all observations in *Y*_*t*_ while $F_{t}^{2}$ makes a 10% under-estimate. The results in Fig 2 show that all the accuracy measures except sMAPE have given the same error for $F_{t}^{1}$ and $F_{t}^{2}$. sMAPE produces a larger error for $F_{t}^{2}$ which indicates it puts a heavier penalty on under-estimates than on over-estimates.

Davydenko and Fildes [24] suggested another scenario to examine the property of symmetry for measures. In this scenario, the reward given for improving the benchmark is expected to balance the penalty given for reducing the benchmark by the same quantity. We also use this to examine our measure UMBRAE. Suppose that a time series has only two observations (*y*) and there is one forecasting method to be compared with another benchmark method. For the benchmark method, it makes the forecasts *f* with errors (*y*−*f*) of 1 and 2 respectively. In contrast, the forecasting method produces errors of 2 and 1 respectively. As an expected result, the forecasting method has an error of 1 measured by UMBRAE based on the benchmark method. Thus, UMBRAE is also symmetric for this case.

**Fig 3:**
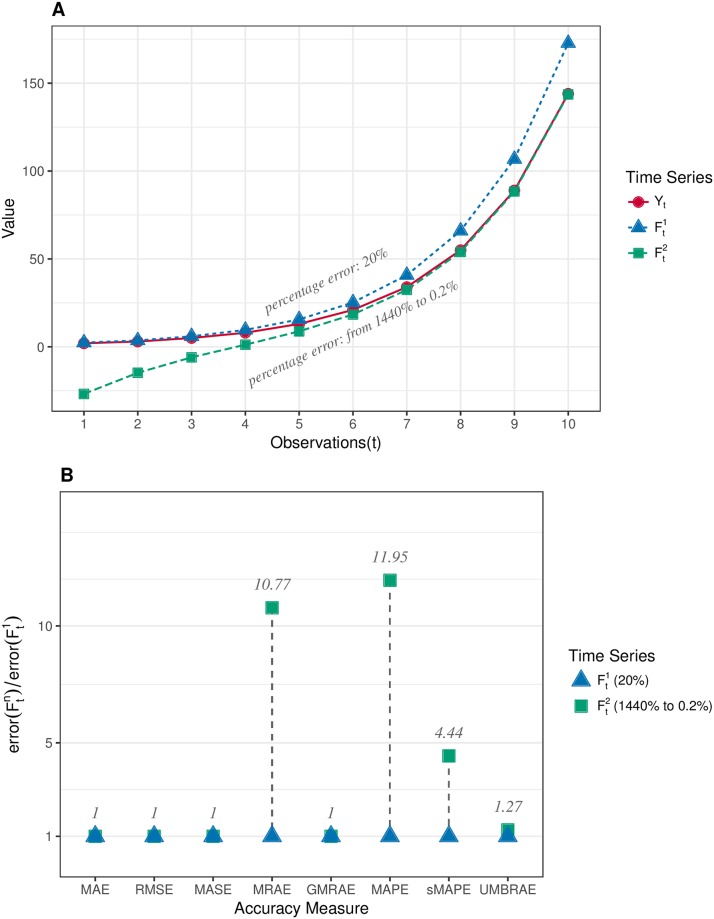
Evaluation on the scale dependency of accuracy measures. A: Synthetic time series data where *Y*_*t*_ is the target series and $F_{t}^{n}$ are forecasts. $F_{t}^{1}$ and $F_{t}^{2}$ have the same mean absolute error, but errors are on different percentage scales to the corresponding values of *Y*_*t*_. B: Results of scale dependency evaluation, where MAE, RMSE, MASE and even GMRAE show no difference between $F_{t}^{1}$ and $F_{t}^{2}$. MRAE and MAPE produce substantially different errors for the two cases. sMAPE and UMBRAE can reasonably distinguish the two forecasts.

Normally, the scale-dependent issue of accuracy measures is related to their capability of evaluating forecasting performance across data series on different scales. Accuracy measures based on percentages or relative ratios are clearly suited to perform such evaluations and no synthetic data are made for this. However, the scale-dependent issue also exists within a data series. Thus, the third group of synthetic data shown in Fig 3 is made to evaluate the property of accuracy measures dealing with data on different scales within a single time series. In this data set, *Y*_*t*_ is a time series generated by the Fibonacci sequence from 2 to 144. As the forecasts to *Y*_*t*_, all forecasting values of $F_{t}^{1}$ are set to have a 20% over-estimate error of the relevant observation of *Y*_*t*_. In contrast, $F_{t}^{2}$ has the same mean absolute error as $F_{t}^{1}$ but its errors are on different percentage scales from 1440% to 0.2%. Specifically, $F_{n}^{2}$ has the same absolute error as $F_{11-n}^{1}$. For instance, $F_{1}^{2}$ has the same absolute error as $F_{10}^{1}$ which is 28.8. As presented in Fig 3, MAE, RMSE, MASE and even GMRAE do not show any difference between the two forecasts. MRAE and MAPE, however, have produced substantially different results for the two cases. The errors measured by them for $F_{t}^{2}$ are approximately ten times larger than for $F_{t}^{1}$. In contrast, UMBRAE and sMAPE give a moderate difference for the two forecasts.

### Evaluation with the M3-Competition data

The M-Competitions are well-known empirical studies which employ various real-world time series data in comparing the performance of forecasting methods. In this study, we use the M3-Competition [7] Data which contains 3003 time series to evaluate our proposed measure. The forecasting data are available with R package ‘Mcomp’ maintained by Hyndman. The ‘Mcomp’ package for R is available from Hyndman’s website: http://robjhyndman.com/software/mcomp/. Among the 24 forecasting methods in the M3-Competition, 22 are used in our evaluation since their forecasts are available for all the 3003 time series. Since the one-step naïve method is used by many accuracy measures as the benchmark, it is also listed in the results as a forecasting method. As an alternative version of MASE, AvgRelMAE which use geometric mean to average errors across time series, is also included in this evaluation. To simplify the results, errors are only measured at the first six forecasting horizons across the 3003 time series, which are available from all of the 22 forecasting methods.

The results are listed in Table 1. It can be noticed that errors by MAE and RMSE are relatively large numbers which is meaningless without comparisons. UMBRAE is able to give interpretable results where a forecasting method with an *error* < 1 can be considered to be better than the benchmark method in terms of the average relative absolute error based on BRAE. As shown in the results, the naïve method, which is the benchmark used by UMBRAE, has an error of 1. Errors of other forecasting methods measured by UMBRAE are all less than 1. This indicates that these forecasting methods are better than the naïve method. However, MRAE gives the opposite result in which the naïve method is ranked as the best. It has to be noticed that all the errors excluding that for the naïve method measured by AvgRelMAE are smaller than 1, whereas all the errors measured by MASE are much larger than 1. The rank correlation coefficient of different measures is shown in Table 2. The correlation between RMSE, or MRAE, and other measures is extremely low. In contrast, UMBRAE shows substantially high agreement with most of other measures, where the average Spearman rank correlation is 0.516. Particularly, UMBRAE has remarkably high correlations with GMRAE and AvgRelMAE which are 0.995 and 0.990 respectively.

**Table 1:** Results on M3-Competition data at first six forecasting horizons.

Forecasting Method	Accuracy Measure																	
	MAE		RMSE		MASE		AvgRelMAE		MRAE		GMRAE		MAPE		sMAPE		UMBRAE	
	Error	Rank	Error	Rank	Error	Rank	Error	Rank	Error	Rank	Error	Rank	Error(%)	Rank	Error(%)	Rank	Error	Rank
**THETA**	589.2	2	1452	11	**1.742**	**1**	**0.7527**	**1**	2.06	5	**0.7186**	**1**	14.85	2	**11.49**	**1**	**0.7882**	**1**
**ForecastPro**	612.3	6	1720	15	1.821	6	0.7556	2	2.62	16	0.7212	2	15.28	4	11.72	2	0.7948	2
**ForcX**	**577.0**	**1**	1269	5	1.768	2	0.7560	3	2.22	9	0.7274	3	**14.81**	**1**	11.72	2	0.8028	3
**COMB S-H-D**	609.1	5	1477	12	1.775	3	0.7784	5	1.86	4	0.7508	5	15.70	8	12.02	4	0.8106	4
**DAMPEN**	630.2	10	1779	16	1.817	5	0.7767	4	2.20	8	0.7473	4	15.85	10	12.05	5	0.8132	5
**AutoBox2**	599.7	3	1190	3	*2.151*	*23*	0.7887	6	2.48	13	0.7557	6	15.86	11	12.47	10	0.8216	6
**PP-Autocast**	640.9	13	1795	19	1.934	13	0.7941	7	2.37	10	0.7633	8	15.78	9	12.28	7	0.8266	7
**HOLT**	667.9	18	1822	20	1.899	10	0.7953	8	2.77	17	0.7616	7	16.87	17	13.16	17	0.8270	8
**B-J auto**	642.1	14	1789	17	1.881	8	0.7957	9	2.40	11	0.7711	10	16.34	13	12.39	9	0.8295	9
**WINTER**	724.5	21	*7326*	*23*	1.997	17	0.8062	10	2.54	14	0.7719	11	18.20	22	13.26	18	0.8340	10
**Auto-ANN**	601.5	4	1180	2	1.896	9	0.8075	12	2.58	15	0.7687	9	15.15	3	12.47	10	0.8386	11
**ARARMA**	*746.4*	*23*	4022	22	1.967	16	0.8138	14	3.03	22	0.7751	12	17.47	20	12.91	16	0.8408	12
**Flors-Pearc1**	656.6	15	1790	18	1.877	7	0.8124	13	2.43	12	0.7816	13	16.75	15	12.78	14	0.8412	13
**ROBUST-Trend**	669.7	19	1651	14	1.806	4	0.8074	11	2.16	7	0.7866	14	18.01	21	13.49	19	0.8500	14
**SMARTFCS**	631.2	11	1496	13	1.920	12	0.8161	15	2.80	18	0.7936	15	15.70	8	12.32	8	0.8552	15
**AutoBox3**	664.3	17	1437	10	1.953	14	0.8335	16	3.01	21	0.8121	16	16.60	14	13.62	21	0.8668	16
**THETAsm**	620.0	9	1264	4	1.901	11	0.8407	17	2.14	6	0.8147	17	15.37	5	12.80	15	0.8689	17
**AutoBox1**	728.7	22	2506	21	2.093	21	0.8465	18	*3.21*	*23*	0.8151	18	17.40	19	13.64	22	0.8721	18
**RBF**	615.7	8	1329	8	2.016	19	0.8496	20	2.96	20	0.8351	19	15.67	6	12.26	6	0.8817	19
**Flors-Pearc2**	635.1	12	1287	6	1.964	15	0.8465	18	2.87	19	0.8361	20	16.82	16	12.76	13	0.8831	20
**SINGLE**	615.3	7	**1157**	1	2.000	18	0.8831	21	1.47	3	0.8713	21	16.09	12	12.68	12	0.9098	21
**naïve 2**	659.8	16	1306	7	2.035	20	0.9054	22	1.41	2	0.9037	22	17.20	18	13.55	20	0.9314	22
**naïve**	719.1	20	1412	9	2.134	22	*1.0000*	*23*	**1.00**	1	*1.0000*	*23*	*18.91*	*23*	*14.69*	*23*	*1.0000*	*23*

The bold numbers highlight the best performance and the italic numbers show the worst. The calculation of MRAE and GMRAE does not include the observations where the naïve error is zero. Zero relative absolute errors are trimmed for GMRAE.

**Table 2:** Spearman’s rank correlation coefficient of the rankings in Table 1.

**Accuracy Measure**	**MAE**	**RMSE**	**MASE**	**AvgRelMAE**	**MRAE**	**GMRAE**	**MAPE**	**sMAPE**	**UMBRAE**
**MAE**	−	0.650	0.409	0.463	0.278	0.493	0.895	0.821	0.476
**RMSE**	0.650	−	−0.110	−0.254	0.357	−0.229	0.449	0.168	−0.260
**MASE**	0.409	−0.110	−	0.711	0.220	0.687	0.510	0.590	0.679
**AvgRelMAE**	0.463	−0.254	0.711	−	0.054	0.985	0.481	0.687	0.990
**MRAE**	0.278	0.357	0.220	0.054	−	0.010	0.079	0.110	0.026
**GMRAE**	0.493	−0.229	0.687	0.985	0.010	−	0.532	0.702	0.995
**MAPE**	0.895	0.449	0.510	0.481	0.079	0.532	−	0.841	0.513
**sMAPE**	0.821	0.168	0.590	0.687	0.110	0.702	0.841	−	0.706
**UMBRAE**	0.476	−0.260	0.679	0.990	0.026	0.995	0.513	0.706	−
**Average**	0.561	0.096	0.462	0.515	0.142	0.522	0.538	0.578	0.516

To eliminate the influence of outliers and extreme errors, we also use trimmed means to evaluate the accuracy measures. A 3% trimming level is used in our study. As shown in Table 3, most errors measured by MAE, RMSE, MASE, MRAE and MAPE have significant differences compared to that without trimming shown in Table 1. The rankings of forecasting methods made by these measures also have significant changes. In contrast, errors and rankings measured by other measures have less changes. Particularly, the value of UMBRAE is quite invariant to trimming, where differences appear only after the third decimal point for most of the forecasting methods. It can also be noticed that the rankings made by UMBRAE in Table 3 keep the same as that in Table 1. In general, all the measures except MRAE have similar rankings. As shown in Table 3, the rank correlations between UMBRAE and other measures are much higher on average as shown in Table 4.

**Table 3:** Results with a 3% trimming level on M3-Competition data at first six forecasting horizons.

Forecasting Method	Accuracy Measure																	
	MAE		RMSE		MASE		AvgRelMAE		MRAE		GMRAE		MAPE		sMAPE		UMBRAE	
	Error	Rank	Error	Rank	Error	Rank	Error	Rank	Error	Rank	Error	Rank	Error(%)	Rank	Error(%)	Rank	Error	Rank
**THETA**	**489.8**	**1**	805.5	2	**1.549**	**1**	**0.7410**	**1**	1.38	8	**0.7360**	**1**	**10.49**	**1**	**10.18**	**1**	**0.7825**	**1**
**ForecastPro**	496.6	3	820.6	3	1.582	3	0.7424	2	1.40	10	0.7395	2	10.79	2	10.37	2	0.7893	2
**ForcX**	494.5	2	**802.1**	**1**	1.577	2	0.7456	3	1.33	05	0.7477	3	10.80	3	10.46	3	0.7977	3
**COMB S-H-D**	508.7	4	831.0	4	1.583	4	0.7662	5	1.28	4	0.7628	5	11.19	4	10.70	5	0.8055	4
**DAMPEN**	509.8	5	840.3	5	1.592	5	0.7653	4	1.35	06	0.7662	7	11.28	6	10.69	4	0.8081	5
**AutoBox2**	520.8	7	842.8	7	1.599	6	0.7776	6	1.50	14	0.7605	4	11.62	11	11.06	11	0.8166	6
**PP-Autocast**	518.6	6	856.6	9	1.629	7	0.7796	7	1.44	12	0.7716	8	11.38	10	10.86	6	0.8218	7
**HOLT**	541.7	14	905.5	16	1.674	12	0.7803	8	1.67	18	0.7656	6	11.97	14	11.42	14	0.8221	8
**B-J auto**	524.3	9	860.7	10	1.653	9	0.7834	9	1.36	7	0.7736	9	11.67	12	11.04	10	0.8248	9
**WINTER**	544.4	16	909.0	17	1.682	14	0.7903	10	1.66	16	0.7786	10	12.06	15	11.48	18	0.8293	10
**Auto-ANN**	523.6	8	841.0	6	1.681	13	0.7947	11	1.54	15	0.7909	11	11.21	5	11.01	8	0.8342	11
**ARARMA**	547.5	17	913.4	18	1.673	11	0.7986	13	1.74	20	0.7943	13	12.17	18	11.42	14	0.8362	12
**Flors-Pearc1**	539.3	13	889.8	15	1.661	10	0.8002	14	1.40	10	0.8002	14	12.15	17	11.40	13	0.8367	13
**ROBUST-Trend**	567.6	20	955.3	21	1.635	8	0.7971	12	1.47	13	0.8080	15	12.62	21	11.87	19	0.8458	14
**SMARTFCS**	529.9	10	862.3	11	1.715	16	0.8047	15	1.67	18	0.7937	12	11.29	8	11.01	8	0.8511	15
**AutoBox3**	566.2	19	945.7	20	1.749	17	0.8215	16	1.81	21	0.8167	16	12.28	19	11.94	20	0.8628	16
**THETAsm**	538.8	12	870.7	13	1.713	15	0.8287	17	1.40	10	0.8359	18	11.31	9	11.37	12	0.8652	17
**AutoBox1**	576.8	22	972.0	22	1.812	20	0.8320	18	*1.85*	*23*	0.8349	17	12.59	20	12.07	21	0.8682	18
**RBF**	533.2	11	863.7	12	1.827	21	0.8369	19	1.83	22	0.8550	20	11.28	6	10.98	7	0.8781	19
**Flors-Pearc2**	555.1	18	887.2	14	1.785	18	0.8374	20	1.69	19	0.8370	19	12.12	16	11.45	17	0.8796	20
**SINGLE**	543.6	15	854.5	8	1.808	19	0.8670	21	1.06	3	0.8947	21	11.83	13	11.43	16	0.9089	21
**naïve 2**	576.1	21	924.0	19	1.844	22	0.8870	22	1.02	2	0.9256	22	12.63	22	12.14	22	0.9323	22
**naïve**	*633.9*	*23*	*1001.9*	*23*	*1.944*	*23*	*1.0000*	*23*	**1.00**	1	*1.0000*	*23*	*14.02*	*23*	*13.26*	*23*	*1.0000*	*23*

The bold numbers highlight the best performance and the italic numbers show the worst.

**Table 4:** Spearman’s rank correlation coefficient of the rankings in Table 3.

**Accuracy Measure**	**MAE**	**RMSE**	**MASE**	**AvgRelMAE**	**MRAE**	**GMRAE**	**MAPE**	**sMAPE**	**UMBRAE**
**MAE**	−	0.951	0.805	0.828	0.256	0.820	0.940	0.970	0.839
**RMSE**	0.951	−	0.707	0.710	0.345	0.708	0.929	0.909	0.720
**MASE**	0.805	0.707	−	0.952	0.239	0.912	0.631	0.751	0.948
**AvgRelMAE**	0.828	0.710	0.952	−	0.135	0.980	0.688	0.771	0.996
**MRAE**	0.256	0.345	0.239	0.135	−	0.077	0.143	0.156	0.133
**GMRAE**	0.820	0.708	0.912	0.980	0.077	−	0.684	0.753	0.985
**MAPE**	0.940	0.929	0.631	0.688	0.143	0.684	−	0.950	0.697
**sMAPE**	0.970	0.909	0.751	0.771	0.156	0.753	0.950	−	0.781
**UMBRAE**	0.839	0.720	0.948	0.996	0.133	0.985	0.697	0.781	−
**Average**	0.801	0.747	0.743	0.758	0.186	0.740	0.708	0.755	0.762

**Fig 4:**
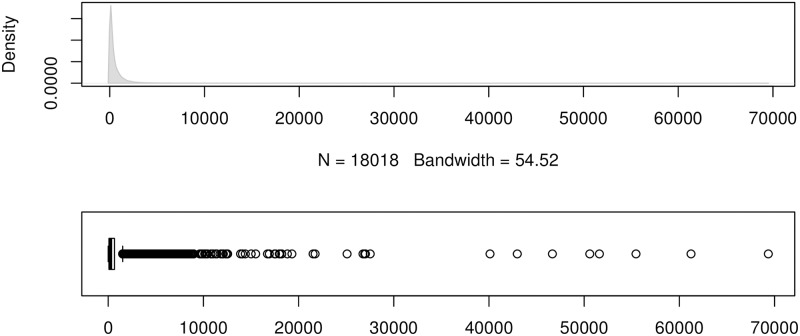
Box-and-whisker plot and kernel density estimates for the absolute errors used by MAE.

**Fig 5:**
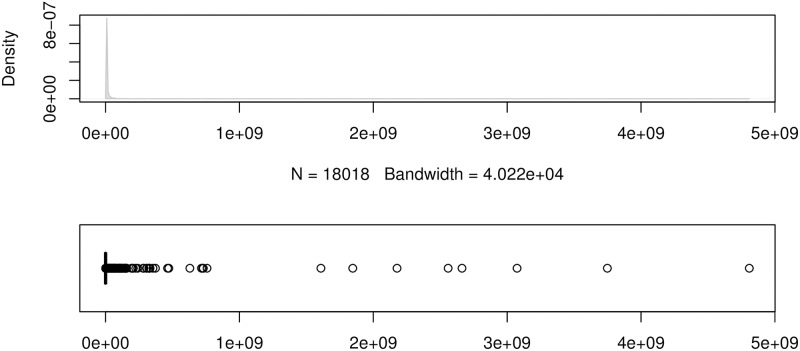
Box-and-whisker plot and kernel density estimates for the squared errors used by RMSE.

**Fig 6:**
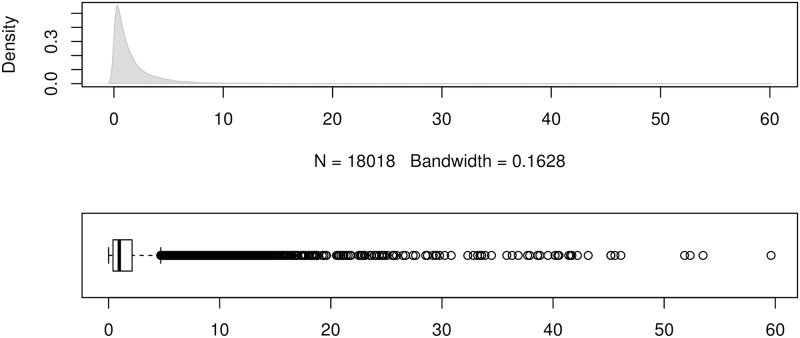
Box-and-whisker plot and kernel density estimates for the absolute scaled errors used by MASE.

**Fig 7:**
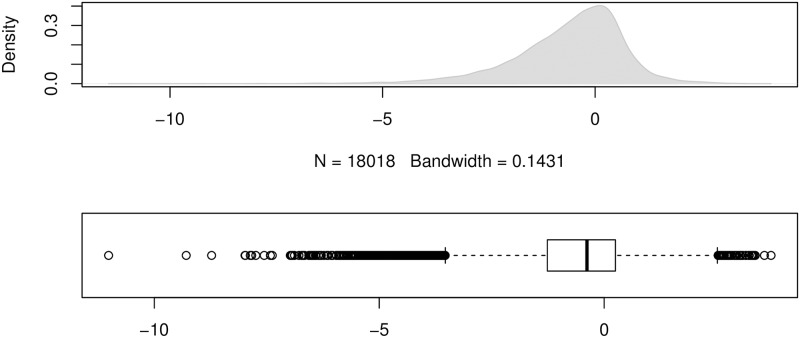
Box-and-whisker plot and kernel density estimates for the absolute scaled errors used by AvgRelMAE (log-scale).

**Fig 8:**
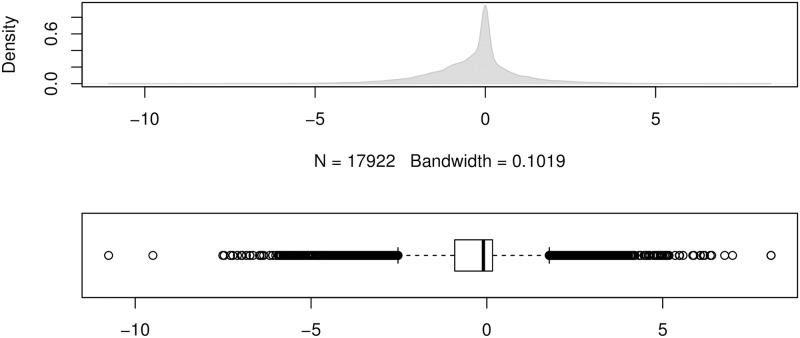
Box-and-whisker plot and kernel density estimates for the relative absolute errors used by MRAE and GMRAE (log-scale, forecasts with zero or undefined error excluded).

**Fig 9:**
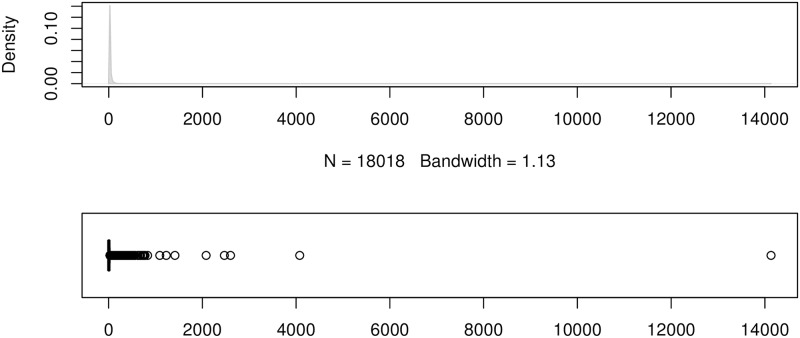
Box-and-whisker plot and kernel density estimates for the absolute percentage errors used by MAPE.

**Fig 10:**
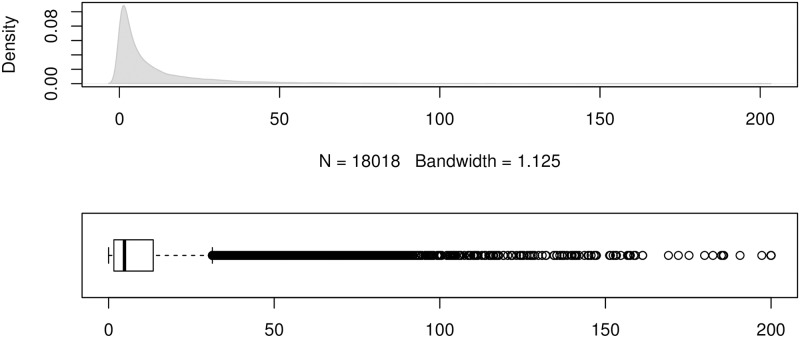
Box-and-whisker plot and kernel density estimates for the scaled percentage errors used by sMAPE.

**Fig 11:**
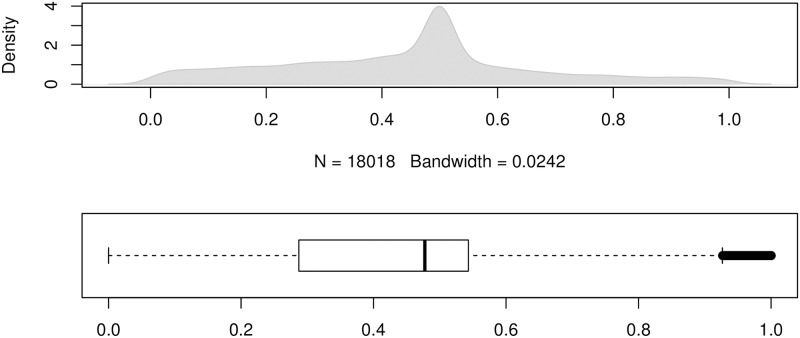
Box-and-whisker plot and kernel density estimates for the bounded relative absolute errors used by UMBRAE (using the naïve errors as the benchmark).

To show the error distributions in a similar manner to that in [24], we use the errors produced by the forecasting method ForecastPro as an example. Figs 4 to 11 show the distributions of the eight underlying error measurements used in the nine accuracy measures mentioned in this paper. In each Fig, the top plot shows the kernel density estimate of the errors illustrating its distribution, while the bottom shows a box-and-whisker plot which more clearly highlights the outliers. From these Figs, it can be seen that the distribution of error measurements used in UMBRAE is more evenly distributed, with fewer outliers than in the other measures.

## Discussion

Fig 1 shows that MRAE and MAPE can be easily dominated by a single forecasting outlier. This is because they are based on the arithmetic mean and there are no upper bound defined for the single error. In practice, the poor resistance to forecasting outliers may produce misleading results. This can be illustrated by our evaluation on the M3-Competition data. As shown in Table 1, MRAE gives significantly different rankings from other measures. It suggests the naïve method performs the best while almost all the other accuracy measures indicate that the naïve method is the worst. By examining the forecasting data, we can find that the results measured by MRAE are seriously distorted by the extreme large relative absolute errors where the naïve errors are small. With the geometric mean, GMRAE has shown remarkable resistance to the forecasting outliers. However, one disadvantage of measures based on the geometric mean is that zero-error forecasts have to be excluded. Thus, these measures may not be sufficiently informative. In contrast, due to the bounded errors defined, we have shown that UMBRAE can perform as well as GMRAE in resisting forecasting outliers. In fact, the errors and rankings given by UMBRAE are remarkably correlated to which measured by GMRAE, especially in Tables 3 and 4 where extreme errors are trimmed. Thus, for the cases where measures such as GMRAE are preferred, UMBRAE could be an alternative measure since it is much easier to use without the need to trim errors.

It can also be noticed in Figs 4 to 11 that all the accuracy measures except AvgRelMAE (see Fig 7), GMRAE (see Fig 8) and UMBRAE (see Fig 11) have highly skewed distributions with long tails including extremely large forecasting outliers. Although undefined and zero errors (0.5%) have been trimmed, GMRAE still contains about 10.2% forecasting outliers including some large log-transformed errors such as -10.76 and 8.08. Although the bounded errors used by sMAPE (see Fig 10) and UMBRAE also contain some outliers, there are no extremely large errors. Specifically, UMBRAE follows a symmetric distribution and it only produces about 3% outliers which will not affect the result significantly.

It has to be noted that UMBRAE does not necessarily always provide the same information as GMRAE. For example, given a time series with a million observations, if the forecasting method and the benchmark method produces errors (*y*−*f*) which are *e* and e* following the standard normal distribution, UMBRAE and GMRAE will both be approximately 1. However, if the forecasting method produces errors of 2*e*, the value of GMRAE will be approximately 2 as one may expected. But, UMBRAE will give an error of approximately 1.67 which is less than 2. This is because the bounded error $\frac{\left|e\right|}{\left|e|+\right|e^{*}|}$ used by UMBRAE will not be increased too much when error *e* is doubled for the cases where |*e*| is much larger than |e*|. In other words, a twice worse forecast will not be given an error of twice in significance by UMBRAE when the forecast is much worse than most of other forecasts. In fact, this is the key strategy of UMBRAE for resisting outliers. Also, the above expectation of error 2 is based on the estimation by ‘relative average error’. However, it is arguable the ‘average relative error’ is not necessarily the same as the ‘relative average error’. This can be more or less reflected by the synthetic test shown in Fig 3. More discussions about this will be given later in this section in terms of the scale-independency. We believe that the above issue does not invalidate the use of UMBRAE in practice.

One of the common concerns about an accuracy measure is whether it is symmetric. Two different cases were used to evaluate the property of symmetry for accuracy measures. In our point of view, the first case is about the symmetry in the absolute quantity which concerns whether the same over-estimates and under-estimates can be treated fairly by a measure. As shown in Fig 2, only sMAPE is not symmetric in the absolute quantity (due to the asymmetric bounded errors used). This issue has been addressed by UMBRAE with symmetric bounded errors defined. The second case is in fact about the symmetry in the relative quantity where measures are expected to give a result of 1 for averaging two relative errors *N* and $\frac{1}{N}$. Normally, a measure which uses the arithmetic mean should not be symmetric in such relative quantity. However, UMBRAE, which uses the arithmetic mean for part of its calculations, has shown a symmetric result. This is because UMBRAE does not work directly on the original error ratios. The original relative errors have been converted to bounded relative errors for UMBRAE before calculating the arithmetic mean. In fact, this is quite similar to the process of calculating GMRAE which is based on the geometric mean. As a result, it is not an issue for UMBRAE to use the arithmetic mean. Figs 8 and 11 show that both errors used by GMRAE and UMBRAE follow a symmetric distribution.

It is necessary (or, at least, highly desirable) for an accuracy measure to be scale-independent when assessing forecasting methods across data on different scales. Normally, measures based on percentages or ratios in the same range are considered to be scale-independent. However, we argue that it is not enough for these percentages or ratios to be in the same range. To be truly scale-independent, these error percentages or ratios should also be closely related to the scale of data for specific observations. Otherwise, they may lead to misleading results. For example, in Table 1, the error of MASE for the naïve method is 2.134. This is a somewhat confusing result which may be intuitively interpreted as indicating that the naïve method performs worse than the naïve method itself! In fact, it means the naïve method gives smaller errors on average for the forecasting data than its errors for the *in-sample* data. In contrast, AvgRelMAE does not have this issue since it uses the average error on *out-of-sample* as the scaling factor. Fig 3 shows that MASE fails to distinguish the difference between the two forecasts which are clearly different considering the error percentages at different observations. This is because every single error used by MASE at different observations is scaled by the same scaling factor. GMRAE also fails in this evaluation. We notice that this is because GMRAE, in fact, has the same issue as MASE. Every single error of GMRAE can also be considered to be a scaled error based on a consistent scaling factor GMAE*, which is the geometric mean of the benchmark errors e*. According to the above, we conclude that MASE, AvgRelMAE and GMRAE are relatively scale-independent because they assume that the scaling factor is a consistent estimator. In contrast, UMBRAE is scale-independent and it is closely related to the error ratios at observations. Thus, it can reasonably show the difference between the two forecasts with respect to error percentages.

Another important property of an accuracy measure is its interpretability. As Table 1 shows, the numerical errors measured by MAE and RMSE have little intuitive meaning without comparisons, and have therefore been scored as ‘fair’. Comparatively, measures which produce errors in percentages or ratios based on a benchmark are more interpretable. The benchmark used by an accuracy measure is also important for its interpretability. In Table 1, errors measured by MAPE are all small errors around 10%. However, these small errors are less meaningful without comparisons. This is because these small percentages are based on the original values of observations. Thus, they do not necessarily indicate a good performance. In contrast, errors measured by UMBRAE are more interpretable. An error of 0.77 indicates that the forecasting method performs approximately 23% better than the benchmark method.

As shown in Table 5, the accuracy measures are rated by the key criteria concerned in this paper. Measures are considered to be less informative if undefined or zero errors have to be excluded. The property of symmetry is rated in both absolute quantity and relative quantity as discussed above. Measures are rated as relatively scale-independent because they assume that the scaling factor is a consistent estimator. Relative-based accuracy measures are considered to be more interpretable than other measures since they can provide more intuitive results in terms of performance without extra comparisons. sMAPE is rated as poor in interpretability since its error, which has a range of (0,200), is not as easy as MAPE to understand.

**Table 5:** Ratings of accuracy measures.

Accuracy Measure	Informative	Resistant to Outliers	Symmetric (absolute,relative)	Scale-independent	Interpretability
**MAE**	good	fair	yes,no	no	fair
**RMSE**	good	poor	yes,no	no	fair
**MASE**	good	fair	yes,no	relatively	good
**AvgRelMAE**	good	good	yes,yes	relatively	good
**MRAE**	fair	fair	yes,no	yes	good
**GMRAE**	fair	good	yes,yes	relatively	good
**MAPE**	fair	fair	yes,no	yes	fair
**sMAPE**	good	good	no,no	yes	poor
**UMBRAE**	good	good	yes,yes	yes	good

In summary, we show that UMBRAE (i) is informative and uses all available errors; (ii) can perform as well as GMRAE in resisting forecasting outliers without the need to trim zero-error forecasts; (iii) is symmetric in both absolute quantity and relative quantity; (iv) is scale-independent; (v) is interpretable and can provide intuitive result. As such, UMBRAE combines the best features of various alternative measures into a single new measure. Thus, we believe UMBRAE is an interesting new measure because it constitutes a simple, flexible, easy to use and understand measure that is resistant to outliers. Also, the forecasting benchmark for calculating UMBRAE is selectable, and the ideal choice should be a forecasting method to be outperformed. As a well-known benchmark, the naïve method can be easily applied as a default to show whether a forecasting method is generally good or not.

## Conclusion

We have proposed a new accuracy measure UMBRAE based on bounded relative errors. As discussed in the review of sMAPE, one advantage of the bounded error is that it gives less significance to outliers since it does not have the issue of being excessively large or infinite. Evaluation on the proposed measure along with related measures has been made on both synthetic and real-world data. We have shown that UMBRAE combines the best features of various alternative measures without having their common drawbacks. UMBRAE, with selectable benchmark, can provide an informative and interpretable result based on bounded relative error. It is less sensitive to forecasting outliers than other measures. It is also symmetric and scale-independent. Though it has been commonly accepted that there cannot be any single best accuracy measure, we suggest that UMBRAE is a good choice for general use when evaluating the performance of forecasting methods. Since UMBRAE, in our study, performs similar to GMRAE without the need to trim zero-error forecasts, we particularly recommend UMBRAE as an alternative measure for the cases where GMRAE is preferred.

Although we have shown that UMBRAE has many advantages as described above, its statistical properties have not been well studied. For example, the way how UMBRAE reflects the properties of the distributions of errors is unclear. Moreover, one possible underlying drawback for UMBRAE is that the bounded error used by UMBRAE will reach the maximum value 1.0 when the benchmark error ($Y_{t}-F_{t}^{*}$) is equal to zero even if the forecast is good. This may produce a biased estimate especially when the benchmark method produces a large number of zero errors. Although this drawback may not be relevant for the majority of real-world data, in the future, we would like to address this issue.
